# PD07 - TNF-α and hypoxia alter the expression of ASM cell contractile protein genes

**DOI:** 10.1186/2045-7022-4-S1-P7

**Published:** 2014-02-28

**Authors:** Nikolaos Tsiougkos, Stamatina Tsapournioti, Paschalis-Adam Molyvdas, Efrosyni Paraskeva

**Affiliations:** 1 Laboratory of Physiology, Faculty of Medicine, University of Thessaly, Larissa, Greece

## Background

Recent research on airway smooth muscle (ASM) cell biology and function has significantly broadened our understanding of the role these cells play in asthma. Apart from being structural contractile cells, they also have immunomodulatory properties. Dynamic multifunctional behavior of ASM cells, namely phenotype plasticity and functional diversity, is now-recognized to be directly involved with tissue inflammation that often interrelates with hypoxia in orchestrating airway remodeling and hyperresponsiveness characteristic of asthma.

## Objective

Herein, we sought to investigate the effect of the inflammatory mediator TNF-α and/or hypoxia on the expression of the ASM cell contractile markers SMMHC (smooth muscle myosin heavy chain), MLCK (myosin light chain kinase), α2 αctin and SM22.

## Method

Primary *in vitro* differentiated human bronchial ASM cell cultures were incubated for 24 hours in the presence or absence of TNF-α under normoxia or hypoxia. Total RNA was extracted, reverse transcribed and mRNA expression levels of SMMHC, MLCK, a2 αctin and SM22 were determined by quantitative real-time PCR.

## Results

TNF-α remarkably increased SMMHC mRNA expression under both normoxia and hypoxia. Treatment under hypoxia, with TNF-α, or their combination decreased MLCK and SM22 mRNA levels. In contrast, α2 αctin mRNA levels were not significantly affected under any of these conditions.

## Conclusion

TNF-α and hypoxia appear to alter the expression of human bronchial ASM cell contractile protein genes, providing new insights on the complex role of ASM cells in the pathophysiology of asthma. Remarkable up-regulation of SMMHC mRNA by TNF-α is indicative of the excessive ASM hypertrophy in severe asthma.

